# The quest for synergy between physical exercise and cognitive stimulation via exergaming in people with dementia: a randomized controlled trial

**DOI:** 10.1186/s13195-018-0454-z

**Published:** 2019-01-05

**Authors:** Esther G. A. Karssemeijer, Justine A. Aaronson, Willem J. R. Bossers, Rogier Donders, Marcel G. M. Olde Rikkert, Roy P. C. Kessels

**Affiliations:** 1 Radboud University Medical Center, Donders Institute for Brain Cognition and Behaviour, Department of Geriatric Medicine, Nijmegen, the Netherlands; 2 Radboud University Medical Center, Radboudumc Alzheimer Center, Nijmegen, the Netherlands; 3 Radboud University Medical Center, Donders Institute for Brain Cognition and Behaviour, Department of Medical Psychology, Nijmegen, the Netherlands; 4 BeweegStrateeg, Groningen, the Netherlands; 5 Center for Human Movement Sciences, University Medical Center Groningen, University of Groningen, Groningen, the Netherlands; 6 Radboud University Medical Center, Department for Health Evidence, Nijmegen, the Netherlands; 7 Center for Cognition, Donders Institute for Brain Cognition and Behaviour, Radboud University, Nijmegen, the Netherlands

**Keywords:** Cognition, Dementia, Alzheimer disease, Exercise, Physical activity, Cognitive stimulation, Exergame, Neuropsychological, Randomized controlled trial

## Abstract

**Background:**

Exercise is often proposed as a non-pharmacological intervention to delay cognitive decline in people with dementia, but evidence remains inconclusive. Previous studies suggest that combining physical exercise with cognitive stimulation may be more successful in this respect. Exergaming is a promising intervention in which physical exercise is combined with cognitively challenging tasks in a single session. The aim of this study was to investigate the effect of exergame training and aerobic training on cognitive functioning in older adults with dementia.

**Methods:**

A three-armed randomized controlled trial (RCT) compared exergame training, aerobic training and an active control intervention consisting of relaxation and flexibility exercises. Individuals with dementia were randomized and individually trained three times a week during 12 weeks. Cognitive functioning was measured at baseline, after the 12-week intervention period and at 24-week follow-up by neuropsychological assessment. The domains of executive function, episodic memory, working memory and psychomotor speed were evaluated. Test scores were converted into standardized *z*-scores that were averaged per domain. Between-group differences were analysed with analysis of covariance.

**Results:**

Data from 115 people with dementia (mean (SD) age = 79.2 (6.9) years; mean (SD) MMSE score = 22.9 (3.4)) were analysed. There was a significant improvement in psychomotor speed in the aerobic and exergame groups compared to the active control group (mean difference domain score (95% CI) aerobic versus control 0.370 (0.103–0.637), *p* = 0.007; exergame versus control 0.326 (0.081–0.571), *p* = 0.009). The effect size was moderate (partial η^2^ = 0.102). No significant differences between the intervention and control groups were found for executive functioning, episodic memory and working memory.

**Conclusions:**

To our knowledge, this is the first RCT evaluating the effects of exergame training and aerobic training on cognitive functioning in people with dementia. We found that both exergame training and aerobic training improve psychomotor speed, compared to an active control group. This finding may be clinically relevant as psychomotor speed is an important predictor for functional decline. No effects were found on executive function, episodic memory and working memory.

**Trial registration:**

Netherlands Trial Register, NTR5581. Registered on 7 October 2015.

**Electronic supplementary material:**

The online version of this article (10.1186/s13195-018-0454-z) contains supplementary material, which is available to authorized users.

## Background

The increasing prevalence of dementia greatly impacts healthcare and society, stressing the need for global action [1]. Since there is no cure or effective disease-modifying drug to treat the most common types of dementia to date [1], research should also focus on the development and implementation of non-pharmacological interventions as an alternative or add-on therapy [2]. Previous research has shown that physical exercise improves cognitive performance in older adults without dementia [3], and that physical inactivity during midlife attributes to the risk of dementia [4, 5]. However, research on cognitive effects of physical exercise in older adults with dementia has shown heterogeneous results [6, 7]. It seems that physical exercise alone may not be enough for older adults with dementia to alter or slow down cognitive decline. Previous studies suggest that combining physical exercise with cognitive stimulation may be a more successful strategy [8, 9].

Animal studies have shown that physical exercise can prime the hippocampus to increase neurogenesis elicited by cognitive stimuli [10, 11]. Furthermore, physical exercise combined with environmental enrichment positively affects hippocampal neurogenesis, possibly via separate pathways, with physical exercise influencing the proliferation of neural precursor cells and environmental enrichment fostering survival of newborn neurons [10]. In line with this, a meta-analysis [12] showed significant benefits of combined cognitive and physical interventions on cognitive function in healthy older adults. These beneficial effects significantly exceeded the effects of physical exercise training alone [12]. In addition, we recently performed a meta-analysis in older adults with mild cognitive impairment (MCI) or dementia which showed that combined cognitive and physical exercise interventions improve global cognitive performance [13]. Thus, these studies illustrate the potential of combined interventions in delaying disease progression in persons with MCI or dementia. However, the superiority of combined interventions over single physical exercise and the effects on different cognitive domains in individuals with dementia remain unknown. Hence, the aim of the current study is to investigate the effects of combined cognitive and physical exercise training on different cognitive domains in people with dementia.

Recent advances in technology present the opportunity to combine physical exercise with cognitively challenging tasks in a single session using exergames [14]. Exergaming is defined by “physical exercise interactively combined with cognitive stimulation in a virtual environment” [15]. Exergame training is a physical–cognitive dual-task training, which requires the mental flexibility to switch between concurrent tasks. Mental flexibility is a core component of executive functioning, a set of higher-order cognitive processes also including cognitive inhibition, planning and problem-solving [16]. We therefore hypothesize that exergame training will specifically benefit executive functioning. Previous research has already shown that exergames improve global cognitive function in healthy older adults and in a clinical population of patients with Parkinson’s disease, schizophrenia, multiple sclerosis and MCI, compared to physical exercise training alone [17]. Moreover, older adults were found to enjoy participation in exergames, which may facilitate long-term activity participation [18]. There is also preliminary evidence that exergames are a feasible and enjoyable intervention for people with dementia [19, 20]. To our knowledge, no previous randomized controlled studies have investigated the effect of exergames on cognitive functioning, more specifically on executive functioning, in older adults with dementia.

Previous studies suggest that the gene apolipoprotein E (*APOE*) may be a moderator in the effects of exercise on cognition [21, 22]. *APOE* is a cholesterol carrier and is important for lipid transport and injury repair in the brain [23]. There are three alleles of *APOE*: *ε2, ε3* and *ε4*. Carrying the ε4 allele of *APOE* is a risk factor for Alzheimer’s disease (AD) and carrying the ε2 allele is protective for AD [1]. Results from cohort studies are contradictory, reporting that physical exercise is both protective for cognitive decline in *APOE ε4* carriers [24, 25] as well as lowering the risk of dementia in APOE ε4 non-carriers [26]. Insight into this moderating relationship may contribute to identify people who will benefit most from our exergame intervention.

The primary aim of the current study is to investigate the efficacy of a 12-week exergame training and aerobic training compared to a control group on executive functioning in older adults with dementia. We hypothesize that exergame training results in greater improvement on executive functioning than aerobic training. Secondary aims are: to assess the feasibility of exergames; to compare effects of exergame training with single aerobic training on the cognitive domains of psychomotor speed, episodic memory and working memory; to measure the follow-up effects of exergame training and aerobic training; and to determine whether the cognitive effects of training are modified by the *APOE* ε*4* carrier state.

## Methods

### Study design

The current study was a 12-week single-blind randomized controlled trial (RCT) with two experimental intervention groups and one active control group. Participants were included from January 2016 to September 2017. The Medical Ethics Committee of Radboud University Medical Center in Nijmegen, the Netherlands approved the research protocol, which was published previously [27]. The study was conducted in compliance with Declaration of Helsinki ethical standards. Participants all verbally agreed to participate in the study and gave written informed consent. The trial is registered at the Dutch trial register (http://www.trialregister.nl) with identification number NTR5581.

### Participants and study procedures

Participants were approached via the memory clinic of Radboudumc Alzheimer Center, day care centres for older adults with cognitive disorders, advertisement in local newspapers and word of mouth. Eligibility criteria for inclusion were: clinically confirmed diagnosis of dementia following the DSM-IV criteria [28] (vascular, Alzheimer or mixed type) with a Mini Mental Status Examination (MMSE) [29] score ≥ 17; aged 60 years or older; if using anti-dementia medication, a stable dose for at least 3 months before the start of the trial; and being capable of giving informed consent [30]. Exclusion criteria were: co-morbidity that limited exercising, including severe cardiovascular, musculoskeletal or neurological disease; diagnosis of a depression, bipolar disorder or psychotic disorder at the moment of inclusion; current drug or alcohol dependency; exercising more than five times per week for at least 30 min at a moderate intensity; wheelchair bound; and severe hearing or visual problems that could not be corrected with the use of hearing aids/glasses. When participants were recruited by newspaper advertisement or word of mouth, we confirmed the dementia diagnosis by investigating their medical record before planning a screening visit. The study was conducted in community centres in Nijmegen, the Netherlands. Participants were randomly assigned to one of the intervention groups or the control group by an independent statistician. The minimization method [31] was used to balance groups for gender, severity of cognitive impairment (MMSE ≥ 20 or < 20), use of medication for Alzheimer’s disease, training location and level of education. The Dutch classification of education levels [32] was used to classify the educational attainment of participants as low (levels 1–3), average (levels 4–5) or high (levels 6–7).

### Interventions

The study included three arms: exergame training, aerobic training and active control. Participants in each arm received three training sessions per week for 12 weeks. Training sessions were given on a one-on-one basis, and trained students or research assistants supervised the participants. Adherence to the intervention was calculated by dividing the number of sessions the participant followed by the total number of sessions that were offered.

The exergame training consisted of a combined cognitive–aerobic bicycle training developed by Bike Labyrinth (www.bikelabyrinth.com). The aerobic training component consisted of cycling on a stationary bike, 30–50 min per session. The aerobic exercise was tailored to an individual fitness level and health status, and aimed to achieve an intensity of 65–75% of heart rate reserve after 12 weeks of training [27]. For participants on medication that attenuates heart rate (e.g. beta-blockers), the Borg Rating of Perceived Exertion (RPE) [33] was used to ensure that the intended training intensity was achieved. In addition, the stationary bike was connected to a video screen. Participants followed a route through a digital environment and simultaneously performed cognitive tasks targeting response inhibition, task switching and processing speed. The exergame training consisted of seven different cognitive training levels. The difficulty of the cognitive tasks increased per level to ensure that the training remained cognitively challenging. The exergame training and different training levels are described extensively in our protocol paper [27].

The single aerobic exercise group consisted of cycling on a stationary bike that was not connected to a video screen. The aerobic training was identical to the exergame training already described. Participants in the active control group received training that consisted of relaxation and flexibility exercises with a duration of 30 min and the same frequency as the training regimes of the intervention groups. The exercises required minimal muscle strength and aerobic capacity and were easy to perform. The level of social engagement was similar to the intervention groups.

### Outcomes

Full assessments were carried out before training (T0), after the 12-week training phase (T2) and 12 weeks thereafter at the 24-week follow-up (F1). Intermediate measurements were performed after 6 weeks of training (T1). Trained research assistants with a background in neuropsychology assessed cognitive performance using a test battery that was described previously [27], and they were blinded to group allocation. The primary outcome measure was objective executive functioning, which was measured by four neuropsychological tasks that were averaged into one domain score: a short form of the Trail Making Test part B [34], the abbreviated 5-line Stroop Color Word Test interference score [35, 36], Letter Fluency [37, 38], and the Rule Shift Cards Test [39]. All tests, except for letter fluency, were also administered after 6 weeks (T1). Secondarily, the following cognitive domains were assessed: episodic memory (Location Learning Test—Revised [40]), working memory (WAIS-III Digit Span [41] and WMS-III Spatial Span [42]), and psychomotor speed (short form of Trail Making Test part A [34] and the abbreviated Stroop Color Word Test parts I and II [35]). Only all psychomotor speed tests were also performed after 6 weeks (T1). Tests were categorized into cognitive domains a priori using the conventional classification described by Lezak et al. [43]. In order to calculate domain scores, test scores were converted into *z*-scores based on the mean and standard deviation of the total sample at baseline [44]. Subsequently, these individual test *z*-scores were averaged per domain.

After inclusion, saliva samples were taken with buccal swabs for *APOE* genotyping. Samples were stored at − 20 °C and analysed using real-time polymerase chain reaction (PCR) [45]. This results in different *APOE* gene phenotypes: three homozygous (*ε2/ε2, ε3/ε3, ε4/ε4*) and three heterozygous (*ε2/ε3, ε2/ε4, ε3/ε4*) [45].

### Statistical analysis

Socio-demographic and clinical characteristics at baseline were presented using descriptive statistics. Feasibility measures (e.g. adherence to the exercise programme, measures of exercise intensity and rating of the exercise sessions) were compared between the groups with one-way analysis of variance (ANOVA) and independent-sample *t* test.

To assess the effect of training on cognitive performance in each domain (i.e. executive function, episodic memory, working memory and psychomotor speed), analysis of covariance (ANCOVA) was performed with post-training cognitive domain *z*-scores as dependent variables, baseline *z*-scores as covariates and group (exergame training, aerobic training and active control) as between-subject factors. To specify significant group effects, Bonferroni-corrected post-hoc tests were performed. To investigate follow-up effects of the intervention for each cognitive domain, we used mixed-model ANCOVA. Variables included in the model werecognitive domain *z*-scores at T2 and F1 as dependent variables, group as between-subject factors, time as within-subject factors, and the corresponding baseline measure as covariates. Additionally, a time × group interaction term was added as a fixed effect. To assess a moderating effect of *APOE ε4*, an interaction term between *APOE ε4* and group was added separately as a predictor.

If a participant had missing data because he/she was cognitively incapable to perform a certain test, the worst possible score for this test was awarded. Afterwards, the domain *z*-score was calculated. If there were missing data due to drop-out and the reason for missingness was at random, missing data were substituted using the multiple imputation method. Characteristic variables of the sample, cognitive domain scores at baseline and training group were included in the imputation model. The following imputation settings were used: automatic model setting, 15 iterations and 5 imputations. If a participant had missing data due to drop-out because of cognitive decline, the criterion for missing at random was not fulfilled. Use of multiple imputation would in this case have been inappropriate as violation of the missing at random assumption biases the estimates [46]. We expected that the cognitive decline would be larger in these participants than the mean decline in the entire group, as it was their reason for drop-out. We decided to use a single value imputation approach for these participants, in which we replaced the missing values by a single value, in our case the greatest decline in the group. To prevent imputing non-realistic values, the lowest possible score was used as a cut-off score. We performed additional sensitivity analyses to check whether this alternative method of dealing with missing data influenced our results.

All statistical analyses were performed as intention-to-treat analyses, including all participants irrespective of adherence to intervention. Additionally, we performed per-protocol analyses including only those participants who successfully completed the intervention period and all measurements. SPSS 22 was used for all analyses with α set at 0.05.

## Results

### Patient flow and sample characteristics

**Fig. 1:**
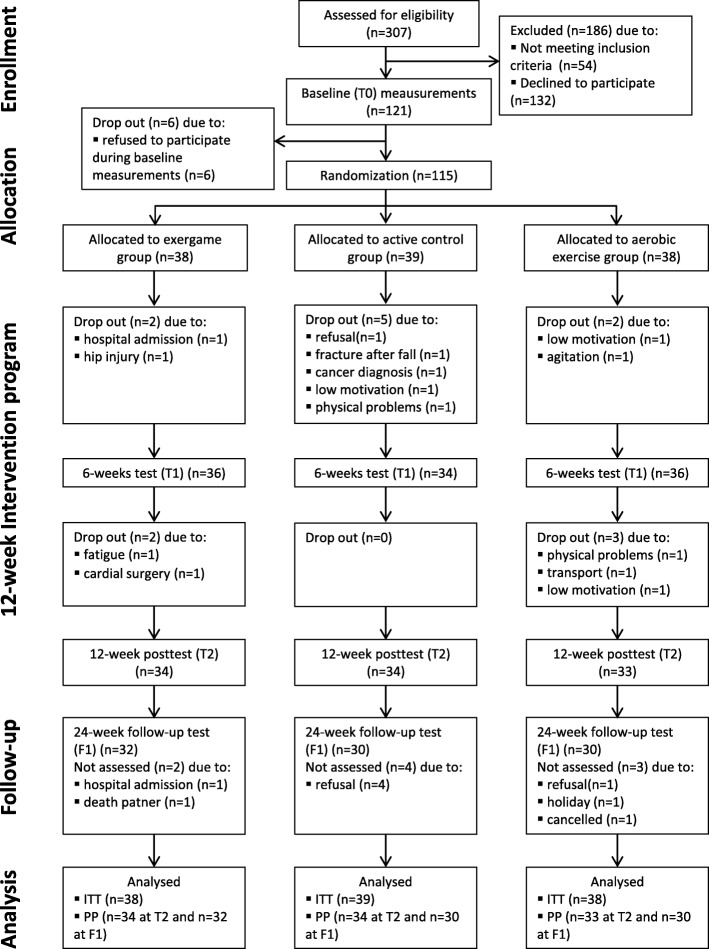
Flowchart of participants in study. ITT intention to treat, PP per protocol

In total, 307 participants were screened for eligibility and 121 participants eventually enrolled in the study. Six participants refused to participate during baseline measurements and the remaining 115 participants were randomized. Fourteen participants did not complete the 12-week intervention (12%). The number of drop-outs did not differ significantly between the groups (*p* = 0.930). The enrolment, allocation process and reasons for drop-out are presented in Fig. 1. Baseline characteristics for the randomized sample were well matched between the groups (Table 1). The included participants had a mean (SD) age of 79.9 (6.5) years and a mean (SD) MMSE score of 22.4 (3.2). There were no differences in age, MMSE score and Katz index between the different dementia types (see Additional file 1).

**Table 1:** Baseline characteristics of the study population

Variable	Exergame group (*n* = 38)	Aerobic group (*n* = 38)	Control group (*n* = 39)
Age (years), mean (SD)	79.0 (6.9)	80.9 (6.1)	79.8 (6.5)
Men, *n* (%)	20 (52.6)	21 (55.3)	21 (53.8)
Educational level, *n* (%)			
Primary school education or lower	6 (15.8)	7 (18.4)	6 (15.4)
Secondary education or vocational training	23 (60.5)	22 (57.9)	22 (56.4)
Higher education	9 (23.7)	9 (23.7)	11 (28.2)
Mini Mental State Examination,^a^ mean (SD)	22.9 (3.4)	22.5 (3.1)	21.9 (3.1)
Aetiology of dementia, *n* (%)			
Alzheimer’s disease	22 (57.9)	16 (42.1)	21 (53.8)
Vascular dementia	4 (10.5)	4 (10.5)	3 (7.7)
Mixed dementia (Alzheimer/vascular)	5 (13.2)	8 (21.1)	11 (28.2)
Not specified	7 (18.4)	10 (26.3)	4 (10.3)
APOE carrier state, *n* (%)			
ε4/ε4	1 (2.7)	5 (13.2)	3 (7.9)
ε3/ε4	20 (54.1)	13 (34.2)	16 (42.1)
ε3/ε3	15 (40.5)	16 (42.1)	16 (42.1)
ε3/ε2	0	3 (7.9)	4 (7.9)
ε2/ε4	1 (2.7)	1 (2.6)	0
ε2/ε2	0	0	0
Duration since dementia diagnosis (months), mean (SD)	13.6 (19.9)	13.8 (12.3)	18.9 (22.4)
Functional Comorbidity Index,^b^ mean (SD)	2.5 (1.9)	2.4 (1.8)	2.2 (1.4)
Katz index,^c^ mean (SD)	5.2 (3.3)	4.5 (3.0)	5.1 (2.9)
Number of medications used, mean (SD)	4.9 (2.9)	5.9 (3.8)	6.1 (3.7)
Use of beta-blockers, *n* (%)	16 (42.1)	17 (44.7)	14 (35.9)
Dementia drugs, *n* (%)			
Rivastigmine	6 (15.8)	4 (10.5)	8 (20.5)
Donezepil	0	0	0
Galantamine	1 (2.6)	3 (7.9)	2 (5.1)
Memantine	0	1 (2.6)	0

*SD* standard deviation

^a^Scores on the Mini-Mental State Examination range from 0 (severe impairment) to 30 (no impairment)

^b^Theoretical range 0–18, higher score indicates more co-morbidities

^c^Theoretical range 0–15, higher score indicates higher dependency in activities of daily living

### Attendance, intensity and safety

Table 2 presents the adherence per group; a trend was found towards higher adherence in the exergame group compared to the aerobic group (mean difference (95% CI) 6.85 (− 0.09 to 13.79), *p* = 0.053). Participants rated both exercise interventions and the active control group highly (see Table 2). Training duration, training load, heart rate and rate of perceived exertion did not differ between both intervention groups. The mean training intensity was light in both intervention groups with an average of 41.8% (SD = 13.3) and 43.5% (SD = 18.2) of maximal heart rate in the exergame group and aerobic group respectively. For the exergame training, the median (interquartile range) training level after 6 weeks was 5.0 (4.3–5.8), and after 12 weeks 5.5 (5.0–6.0). After 6 weeks, 25% of the participants reached level 6 or 7, and 50% reached level 5. After 12 weeks, 50% of the participants reached level 6 or 7, and 40% reached level 5. This demonstrates that there were no floor effects for the cognitive stimulation activity and about half of the participants were able to complete the highest levels, thus showing that the exergame training was feasible and that adequate skill acquisition was present. No occurrence of serious adverse events (e.g. events leading to death, hospital admission or persistent disability) related to the exercise interventions were recorded.

**Table 2:** Training characteristics of the study population

Variable	Exergame group (*n* = 38)	Aerobic group (*n* = 38)	Control group (*n* = 39)
Adherence rate (%), mean (SD)	87.3 (13.6)*	81.1 (13.7)*	85.4 (12.9)
Duration training session (min), mean (SD)	32.6 (6.0)	30.5 (8.7)	30^a^
Training load (W), mean (SD)	53.7 (34.9)	51.2 (27.7)	NA
Resting heart rate (beats/min), mean (SD)	79.4 (12.1)	77.9 (10.4)	NA
Heart rate during training (beats/min), mean (SD)	105.5 (14.8)	103.9 (14.3)	NA
Heart rate difference (beats/min), mean (SD)	26.1 (15.1)	26.0 (13.8)	NA
Training intensity^b^ (% of maximal heart rate), mean (SD)	41.8 (13.3)	43.5 (18.2)	NA
Rate of perceived exertion during training,^c^ mean (SD)	13.1 (1.2)	12.8 (1.9)	NA
Rating of training sessions^d^ (scale 1–5), Median (interquartile range)	5.0 (4.0–5.0)	5.0 (4.0–5.0)	5.0 (4.0–5.0)
Training level after 6 weeks^d^ (scale 1–7), Median (interquartile range)	5.0 (4.3–5.8)	NA	NA
Training level after 12 weeks^d^ (scale 1–7), Median (interquartile range)	5.5 (5.0–6.0)	NA	NA

Differences between groups tested with one-way analysis of variance (three groups) or independent-sample *t* test (two groups), if data were normally distributed. For post-hoc comparisons, Tukey honest significant difference test was performed. If data was not normally distributed, Kruskall Wallis test was performed.

*NA* not applicable, *SD* standard deviation

^a^All training sessions lasted for 30 min, time has not been recorded

^b^Training intensity only calculated for participants who do not use beta-blockers (*n* = 21 and *n* = 20 in the exergame group and the aerobic group respectively)

^c^Theoretical range 6–20, where 6 indicates lowest intensity level and score 20 indicates highest intensity level

^d^Data not normally distributed, therefore presented as median (interquartile range)

*A trend was found towards higher adherence in the exergame group compared to the aerobic group (mean difference (95% confidence interval) 6.85 (− 0.09 to 13.79), *p* = 0.053)

### Missing data

Missing data due to drop-out of participants was 0% at T0, 8.7% at T1, 9.6% at T2 and 17.5% at F1. Reasons for drop-out are described in Fig. 1. In a total of six cases, the reason for drop-out was refused participation (five out of six at follow-up measurements). Reason for refusal was cognitive decline, which led to caregivers’ withdrawal of consent. As explained in Methods, we used single-value imputation for substituting missing data not at random, and performed additional sensitivity analyses to check whether this influenced our results. Data for the remaining eight drop-outs were missing at random and were substituted using multiple imputation, as explained in Methods.

### Intention-to-treat analysis

**Fig. 2:**
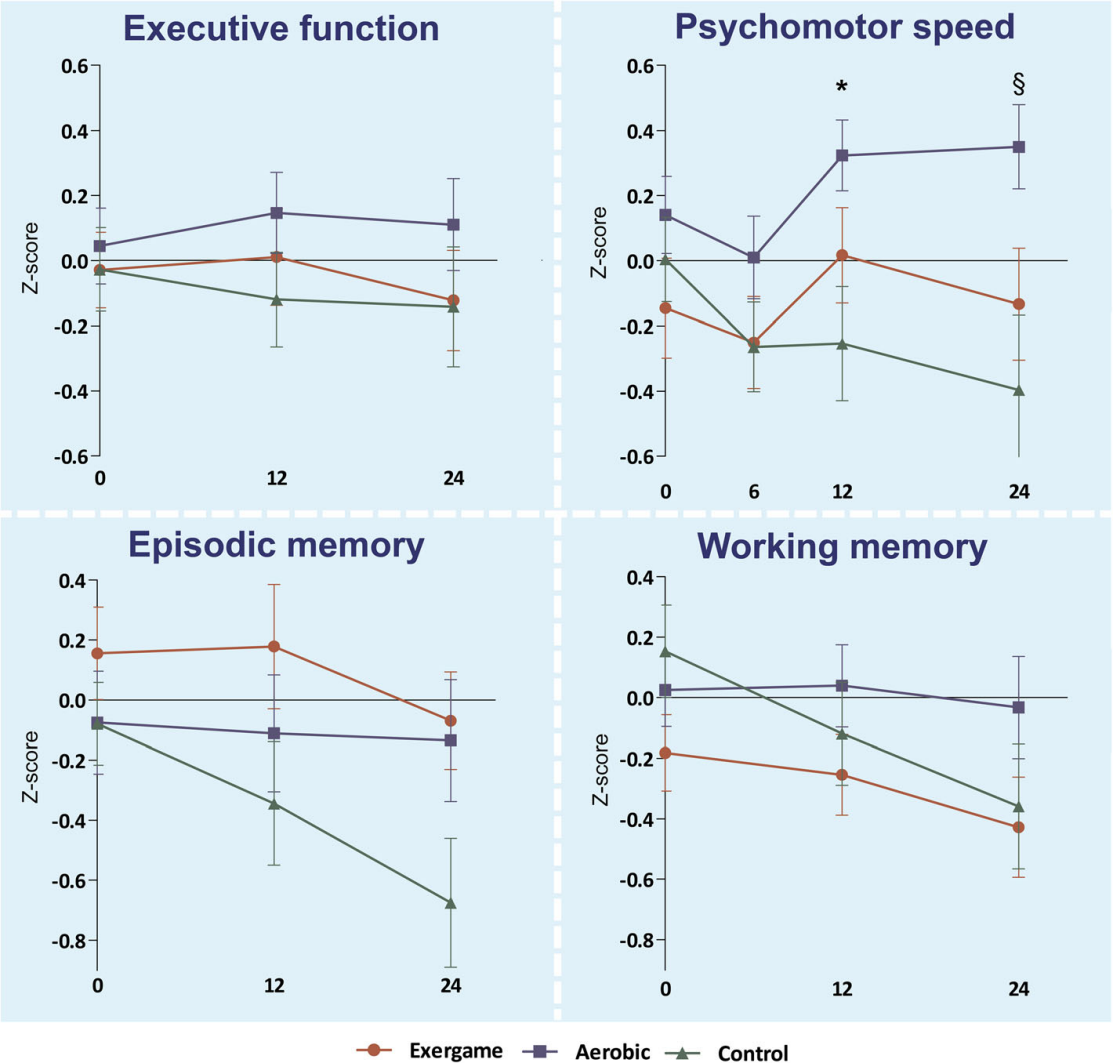
Mean *z*-scores and standard errors of mean (SEM) at baseline, after 12 weeks and after 24 weeks for domains of executive function, psychomotor speed, episodic memory and working memory. Arrows represent SEM. *Significant effect (*p* < 0.05) of exergame training and aerobic training on psychomotor speed compared to controls after 12 weeks; §maintenance effect (*p* < 0.05) of aerobic and exergame training on psychomotor speed at 24-week follow-up

Figure 2 shows the performance on the four cognitive domains at each time point per treatment arm. No significant differences were found between the exergame group, aerobic group and control group on executive functioning after 12 weeks of training. Since after 6 weeks (T1) letter fluency was not administered as an executive function test, we decided not to include T1 data in our analyses. Significant improvement on the secondary measure psychomotor speed was found for both the aerobic and the exergame group compared to the controls after 12 weeks of training (mean difference domain score (95% CI) aerobic versus control 0.370 (0.103–0.637), *p* = 0.007; exergame versus control 0.326 (0.081–0.571), *p* = 0.009). The size of the effect was moderate (partial η^2^ = 0.102). This effect was not yet present at the intermediate measurements after 6 weeks (see Fig. 2). No significant differences were found between the groups on the secondary measures of episodic memory and working memory after the 12-week intervention period. An additional sensitivity analysis yielded similar results, which shows that our findings are robust. Follow-up analysis showed that the improvement in psychomotor speed was maintained for both the aerobic group and the exergame group compared to the controls (mean difference domain score (95% CI) aerobic versus control 0.453 (0.185–0.722), *p* = 0.001; exergame versus control 0.326 (0.070–0.604), *p* = 0.014. There was no significant difference between the exergame and aerobic group (mean difference domain score (95% CI) exergame versus aerobic − 0.116 (0.399 to – 0.398), *p* = 0.399). We did not find any between-group differences in any of the other cognitive domains at follow-up. Sensitivity analysis pointed in the same direction, with a maintenance effect in the aerobic group compared to controls (mean difference domain score (95% CI) aerobic versus control 0.267 (0.048–0.486)), and no follow-up effect in any of the other cognitive domains. Moderator analysis showed that carrying *APOE ε4* did not influence the relation between training and cognitive performance. *z*-scores of the different cognitive domains per group and time point are presented in Additional file 2. Raw data of cognitive test scores are presented in Additional file 3.

### Per-protocol analysis

n the per-protocol analyses, we excluded 14 participants who did not complete the 12-week intervention period. The remaining 101 participants were included in this analysis. The results of the per-protocol analyses were in line with the intention-to-treat analyses, with positive effects of exergame and aerobic training on psychomotor speed compared to controls (mean difference domain score (95% CI) aerobic versus control 0.322 (0.038–0.607), *p* = 0.021; exergame versus control 0.283 (0.002–0.563), *p* = 0.047). As in the intention-to-treat analyses, no significant between-group differences were observed in the domains of executive function, memory and working memory. At follow-up there were nine additional drop-outs, which led to inclusion of 92 participants in the follow-up analysis. We found that there was a trend for maintained improvement in psychomotor speed at 24-week follow-up in the aerobic group compared to the control group (mean difference domain score (95% CI) aerobic versus control 0.267 (0.048–0.486), *p* = 0.057). No significant intervention effects were observed in any of the other domains.

## Discussion

To our knowledge, this is the first randomized controlled trial to investigate the differential effect of exergaming versus aerobic training on cognitive functioning in people with dementia. We hypothesized that exergame training would result in greater improvement on executive functioning than single aerobic training. Although we did not find an effect of exergame training or aerobic exercise on executive function after 12 weeks, we found that psychomotor speed improved in both the exergame and the aerobic group compared to active controls. This effect was maintained at the 24-week follow-up. We did not find an effect of both intervention groups in the cognitive domains of episodic memory and working memory compared to the control group. Moderator analysis showed that *APOE ε4* carriership did not influence the relation between training and cognitive function. Finally, we demonstrated that a newly developed exergame that comprises both physical and cognitive training elements is feasible for people with dementia.

### Interpretation of results and comparison with previous research

Contrary to our hypothesis, the current results did not show a larger effect of exergame training compared to aerobic training on cognitive functioning. Comparable research on the differential effects of combined cognitive and physical training versus only cognitive or physical interventions in people with dementia is scarce. There is one previously published paper reporting that neither a 12-week combined cognitive–aerobic training nor aerobic training only improved global cognitive function in a smaller sample of 80 individuals with AD [47]. However, the type of intervention and used outcome measures are incomparable to the current study. Research in individuals with MCI showed inconsistent findings regarding the cognitive benefits of combined interventions and its potential superiority compared to physical exercise or cognitive training alone [48]. In contrast, for older adults without cognitive impairment there is converging evidence that combined interventions (including exergames) are superior to physical or cognitive training alone [48], with larger effect sizes for interventions that are performed simultaneously compared to sequential interventions [12].

In healthy older adults, evidence for the efficacy of physical exercise and combined cognitive and physical interventions on executive functions [12, 49], memory [12, 49], working memory [12, 50] and attention [51] have been well established. In our current study, both exergame and aerobic-only training did not positively affect executive functions, working memory or episodic memory. This seems partly in line with previous research. A meta-analysis performed by our group [13] demonstrated positive effects of combined interventions on global cognitive function in older adults with MCI or dementia, but no effects in the domains of executive function and memory. In contrast, a recently published RCT showed that both a mentally challenging exergame and a passive exergame improve executive functioning in people with MCI [52]. However, the more challenging exergame only yielded significant effects after 6 months of training, while the passive exergame already produced gains after 3 months [52]. A possible explanation for this discrepancy is that participants in the mentally challenging exergame group needed more time to master the intervention, which may have delayed triggering the synergistic effects of the combined intervention [52]. This might also explain the negative findings in our study, since a mentally challenging exergame was used for a relative short intervention period of 12 weeks.

There is evidence that the severity of neurocognitive disorder has a moderating impact on the cognitive effects of combined cognitive and physical training [53]. An increase in the severity of neurocognitive disorder may lead to a decrease of the intervention effect [53]. This could be explained by a reduced structural brain capacity (e.g. reduced number of neurons and synapses) in participants with more severe neurocognitive disorder, which may lead to limited resources necessary for training-induced gains [53]. Therefore, it may be more difficult to induce cognitive benefits in people with dementia compared to those with MCI or healthy older adults. Moreover, the complexity to obtain valid neuropsychological outcomes that are sensitive to change in persons who already have severe cognitive deficits due to their dementia complicates the assessment of cognitive functioning in this group. Even though we carefully selected and adjusted tests for use in mild-to-moderate dementia, it is particularly challenging to assess executive functions in this group. Executive functions include higher-order processes such as inhibitory control, mental flexibility and planning, which are already affected in the early stages of dementia [54, 55]. Assessment of executive function in people with dementia may consequently result in floor effects or missing data, which make it difficult to measure change over time.

In our study we found a moderate effect of exergame training and aerobic training on psychomotor speed after a 12-week training period in people with dementia. This effect was not yet present after 6 weeks of training. Firstly, this may imply that the improvement is due to the training and not due to non-specific treatment or practice effects. Secondly, this suggests that a longer training duration is necessary to improve psychomotor speed. Although still under debate, there is some evidence that physical exercise leads to improved cognitive function through promotion of hippocampal neurogenesis [56], brain angiogenesis [57] and synaptic plasticity [58] elicited by an increased expression of neurotrophic factors [59]. In cognitively healthy older adults, physical exercise interventions have the largest gains on executive control processes, psychomotor speed and attention [49, 51, 60, 61]. In people with dementia there is little research about the benefits for different cognitive domains. From a neurobiological perspective, however, we do not have an explanation for why exercise would only improve psychomotor speed, but not the other cognitive skills assessed. We hypothesize, that only finding an effect on psychomotor speed, and not on executive functioning, may be related to domain-specific responsiveness of the selected outcome measures. Processing speed tests typically are continuous outcome measures without ceiling or floor effects that are highly sensitive [62], which may explain the sensitivity to change even in a dementia sample. In contrast, tests that measured executive functioning resulted in floor effects in our dementia sample, which made it difficult to measure change over time. Alternatively, one could also hypothesize that mood may be a mediating factor for improvement on speed measures, as previous research showed that exercise and exergame training can reduce depressive symptoms in healthy older adults [63, 64]. The positive effect on psychomotor speed was consistent across the different neuropsychological tests used to measure psychomotor speed (short form of Trail Making Test part A and the abbreviated Stroop Color Word Test parts I and II), which shows that the effect was robust and reliable. Its moderate effect size is slightly larger than to the small-to-moderate effect sizes commonly found in studies examining the effects of cholinesterase inhibitors on cognitive function [65, 66]. Given that interventions to ameliorate cognitive decline of people with dementia are scarce, this effect size may be clinically relevant. Poor processing speed is a predictor of functional decline in basic and instrumental activities of daily living [67]. In addition, poor processing speed is reported to be a predictor for incident dementia [68] and was found to be associated with shorter survival among older adults in Japan [69]. Furthermore, late-life cognitive decline is attributable to slower processing speed [70]. Thus, the reported improvement in processing speed may be clinically relevant.

The mean training intensity was light in both intervention groups, with an average of 41.8% (SD = 13.3) and 43.5% (SD = 18.2) of maximal heart rate in the exergame group and the aerobic group respectively. We expected that improved cardiorespiratory fitness would be a requirement to improve cognitive function [51], and therefore we aimed to achieve moderate exercise intensity (e.g. 65–75% of maximal heart rate) during the training sessions. However, the exercise training was tailored to an individual fitness level and health status, and most participants were not able to achieve a moderate training intensity. The recently published Dementia and Physical Activity (DAPA) trial [71] showed that moderate to high-intensity aerobic and strength exercise training did not slow cognitive decline in people with mild to moderate dementia, and even worsened cognitive impairment in those who complied with the intervention, despite an improvement in physical fitness. It is therefore unlikely that the light training intensity in our study limited the beneficial effects of exercise on cognitive functioning.

### Strengths and limitations

The strengths of our study include the inclusion of a relatively large sample of people with dementia, a high adherence rate, the use of a comprehensive neuropsychological assessment and follow-up measurement for long-term maintenance effects. However, some limitations need to be taken into account when interpreting our results. Firstly, only participants who were mobile and motivated enrolled in our study, which may limit the external validity of the current findings. Secondly, participants were not blinded to allocation, which is an unavoidable limitation of exercise studies. Outcome assessors were masked for intervention allocation. Thirdly, although we used adapted versions of executive tests, making administration in people with dementia more feasible, a floor performance was still found in a number of individuals. This may have reduced the sensitivity to measure change over time, obscuring potential positive results. Fourthly, the intervention period was only 12 weeks, which may have been too short to show beneficial effects of exergames on executive functioning. Lastly, future studies should include measures of mood, since this might be a potential mediating factor for the improvement in processing speed measures.

### Clinical relevance and feasibility

Both exergame training and aerobic training improved psychomotor speed after 12 weeks, with a moderate effect size. This finding may be clinically relevant as psychomotor speed is an important predictor for functional decline. In our study, exergame training was not superior to aerobic training. However, there was a trend for higher adherence in the exergame group compared to the aerobic group. Additionally, trainers who individually guided the training sessions reported that it was easier to motivate participants in the exergame group and to increase duration of the training sessions. This was confirmed by our finding that no participants dropped out in the exergame group due to low motivation (see Fig. 1). Accordingly, exergaming seems to be an effective method to stimulate long-term physical activity participation in people with dementia.

### Future directions

Future studies should examine whether certain individual characteristics (e.g. type of dementia) moderate the effect of physical activity on cognition. Insight into these individual differences is important because it can determine which people are most likely to benefit from physical activity. It can also help to personalize interventions, thereby stimulating physical activity. Moreover, additional studies are needed to explore the optimal intervention design and dose–response for eliciting beneficial cognitive effects in people with dementia. Future intervention trials should include measures of psychomotor speed as these can reliably and validly be assessed in people with dementia and are closely related to everyday activities. Furthermore, studies should also focus on investigating neurophysiological mechanisms that underlie the cognitive effects of exercise, for example by including neuroimaging measures.

## Conclusions

Exergaming is a feasible and highly appreciated exercise method to engage older adults with dementia in physical exercise, mixed with cognitive stimulation. Both exergame training and aerobic training can improve psychomotor speed, which may be clinically relevant as psychomotor speed is an important predictor for functional decline. Although no effects were found on executive function, episodic memory and working memory, the potential broad range of effects of exergames for older adults with dementia (e.g. physical functioning, quality of life, activities in daily living) should be studied in future RCTs.

## Additional files

Additional file 1: Baseline characteristics of study population presented separately for different types of dementia (DOCX 18 kb)

Additional file 2: *z*-scores of different cognitive domains per group and time point. (DOCX 18 kb)

Additional file 3: Data of cognitive tests for each intervention group. (DOCX 21 kb)

## Abbreviations

AD: Alzheimer’s disease
APOE: Apolipoprotein E
MCI: Mild cognitive impairment
MMSE: Mini Mental State Examination
RCT: Randomized controlled trial
