# Supplementary material for: The quest for synergy between physical exercise and cognitive stimulation via exergaming in people with dementia: a randomized controlled trial
Source: Alzheimers Res Ther. 2019 Jan 5;11:3. doi: 10.1186/s13195-018-0454-z (PMC6320611; doi:10.1186/s13195-018-0454-z)
Supplement: Supplementary file 1 — Baseline characteristics of study population presented separately for different types of dementia (DOCX 18 kb) [file 13195_2018_454_MOESM1_ESM.docx]

**Additional file 1:** Baseline characteristics of the study population presented separately for the different types of dementia.

| Variables | | Alzheimer’s dementia  (n=59) | Vascular dementia  (n=11) | Mixed dementia (Alzheimer/Vascular)  (n=24) | Not specified  (N=21) |
| --- | --- | --- | --- | --- | --- |
| Age, years, mean (SD) | | 79.3 (6.9) | 81.5 (5.1) | 79.4 (5.5) | 81.4 (7.0) |
| Men, n (%) | | 28 (47.5) | 7 (63.6) | 17 (70.8) | 10 (47.6) |
| Educational level, n (%) | |  |  |  |  |
| - Primary school education or lower | | 9 (15.3) | 0 (0) | 2 (8.3) | 8 (38.1) |
| - Secondary education or vocational training | | 35 (59.3) | 6 (54.5) | 17 (70.8) | 9 (42.9) |
| - Higher education | | 15 (25.4) | 5 (45.5) | 5 (20.8) | 4 (19.0) |
| Mini Mental State Examination, mean (SD)^a^ | | 21.8 (3.2) | 23.7 (2.2) | 23.1 (3.7) | 22.8 (3.0) |
| APOE ε4 carrier, n (%) |  | 30 (50.8) | 4 (36.4) | 14 (58.3) | 12 (57.1) |
| Functional Comorbidity Index^b^, mean (SD) | | 1.9 (1.7) | 2.9 (1.1) | 2. 4 (1.1) | 3.4 (2.1) |
| Katz index^c^, mean (SD) | | 4.5 (3.1) | 5.4 (2.6) | 5.5 (3.0) | 5.5 (3.5) |
| Number of medication used, mean (SD) | | 4.5 (2.9) | 6.1 (3.3) | 7.8 (4.0) | 5.9 (3.5) |
| Dementia drugs, n (%) | | 21 (35.6) | 0 (0) | 3 (12.5) | 0 (0) |
| Intervention group | |  |  |  |  |
| - Exergame group | | 22 (37.3) | 4 (36.4) | 5 (20.8) | 7 (33.3) |
| - Aerobic group | | 16 (27.1) | 4 (36.4) | 8 (33.3) | 10 (47.6) |
| - Control group | | 21 (35.6) | 3 (27.3) | 11 (45.8) | 4 (19.0) |

^a^ Scores on the Mini-Mental State Examination range from 0 (severe impairment to 30 (no impairment)

^b^ Theoretical range 0–­18 and a higher score indicates more comorbidities

**^c^** Theoretical range 0–15 and a higher score indicates higher dependency in activities of daily living
