# Supplementary material for: The quest for synergy between physical exercise and cognitive stimulation via exergaming in people with dementia: a randomized controlled trial
Source: Alzheimers Res Ther. 2019 Jan 5;11:3. doi: 10.1186/s13195-018-0454-z (PMC6320611; doi:10.1186/s13195-018-0454-z)
Supplement: Supplementary file 2 — z-scores of different cognitive domains per group and time point. (DOCX 18 kb) [file 13195_2018_454_MOESM2_ESM.docx]

**Additional file 1.** Z-scores of the different cognitive domains per group and time point.

|  | Exergame group (n=38) | | | | Aerobic group (n=38) | | | | Control group (n=39) | | | | F test value (df), p-value | |
| --- | --- | --- | --- | --- | --- | --- | --- | --- | --- | --- | --- | --- | --- | --- |
|  | **T0** | **T1** | **T2** | **F1** | **T0** | **T1** | **T2** | **F1** | **T0** | **T1** | **T2** | **F1** | **T0-T2** | **T2-F1** |
| Executive function, mean z-score (SD) | -0.03 (0.71) | Na | 0.01 (0.88) | -0.12 (0.88) | 0.05 (0.72) | Na | 0.15 (0.74) | 0.11 (0.80) | -0.03 (0.80) | Na | -0.12 (0.87) | -0.14 (1.10) | F(2,115) = 1.095, p=0.338 | F(2,103)=0.254, p=0.776 |
| Psychomotor speed, mean z-score (SD) | -0.15 (0.94) | -0.25 (0.84) | 0.02 (0.87) | -0.13 (0.98) | 0.14 (0.73) | 0.01 (0.75) | 0.32 (0.64) | 0.35 (0.73) | 0.00 (0.81) | -0.26 (0.83) | -0.25 (1.04) | -0.39 (1.37) | F(2,115) = 5.772, **p=0.004*** | F(2,103)=6.127, **p=0.003^§^** |
| Episodic memory, mean z-score (SD) | 0.16 (0.95) | Na | 0.18 (1.23) | -0.07 (0.93) | -0.08 (1.05) | Na | -0.11 (1.15) | -0.13 (1.15) | -0.08 (0.86) | Na | -0.34 (1.21) | -0.67 (1.29) | F(2,115) = 1.720, p=0.184 | F(2,101)=2.151, p=0.122 |
| Working memory, mean z-score (SD) | -0.18 (0.77) | Na | -0.25 (0.79) | -0.43 (0.95) | 0.02 (0.73) | Na | 0.04 (0.80) | -0.03 (0.95) | 0.15 (0.95) | Na | -0.12 (1.02) | -0.36 (1.24) | F(2,115) =1.907, p=0.153 | F (2,103)=2.963, p=0.056 |

Abbreviations: Na=not applicable

Values are presented as mean z-score (standard deviation). Differences between groups after the 12-week training period were tested with One-way Analysis of Covariance (ANCOVA). Follow-up effects were tested with mixed-model ANCOVA. **P < 0.05**

**^*^** Bonferroni post-hoc test showed a significant improvement for both the aerobic and the exergame group compared to controls after 12 weeks of training (∆ aerobic *versus* control: 0.370 [CI: 0.103–0.637], p=0.007; ∆ exergame *versus* control: 0.326 (0.081–0.571), p=0.009). § improvement in psychomotor speed was maintained at 24-week follow-up (∆ aerobic *versus* control: 0.453 [CI: 0.185–0.722]; ∆ exergame *versus* control: 0.337 [CI: 0.070–0.604]).
