# Supplementary material for: The quest for synergy between physical exercise and cognitive stimulation via exergaming in people with dementia: a randomized controlled trial
Source: Alzheimers Res Ther. 2019 Jan 5;11:3. doi: 10.1186/s13195-018-0454-z (PMC6320611; doi:10.1186/s13195-018-0454-z)
Supplement: Supplementary file 3 — Data of cognitive tests for each intervention group. (DOCX 21 kb) [file 13195_2018_454_MOESM3_ESM.docx]

**Additional file 3.** Data of cognitive tests for each intervention group presented as mean (standard deviation)

|  |  | T0, pretest | T1, 6-week test | T2, 12-week posttest | F1, 24-week follow-up |
| --- | --- | --- | --- | --- | --- |
| Executive function | | | | | |
| Trail making test part B (sec) | Exergame group | 166.6 (95.1) | 147.1 (98.8) | 160.7 (106.2) | 178.1 (105.4) |
|  | Aerobic group | 155.6 (100.3) | 157.6 (95.4) | 124.9 (97.9) | 141.1 (96.5) |
|  | Control group | 165.8 (101.5) | 172.6 (114.6) | 170.1 (106.8) | 166.1 (105.1) |
| Stroop test speed-accuracy trade off scores | Exergame group | 0.35 (0.17) | 0.36 (0.18) | 0.37 (0.18) | 0.34 (0.18) |
|  | Aerobic group | 0.37 (0.18) | 0.33 (0.15) | 0.37 (0.18) | 0.32 (0.15) |
|  | Control group | 0.32 (0.18) | 0.33 (0.21) | 0.31 (0.18) | 0.29 (0.21) |
| Stroop test colour-word interference card (sec) | Exergame group | 138.2 (72.4) | 132.8 (72.5) | 126.9 (63.7) | 139.2 (75.2) |
|  | Aerobic group | 126.2 (66.2) | 129.6 (71.3) | 121.6 (61.7) | 123.8 (63.5) |
|  | Control group | 155.8 (88.4) | 155.8 (85.7) | 152.5 (81.3) | 136.5 (74.5) |
| Stroop test colour-word interference card (number of errors) | Exergame group | 7.5 (8.5) | 9.0 (13.1) | 8.0 (12.8) | 6.1 (8.8) |
|  | Aerobic group | 5.8 (6.9) | 7.6 (9.6) | 6.0 (8.3) | 6.6 (7.9) |
|  | Control group | 6.4 (8.9) | 8.0 (10.3) | 10.7 (13.5) | 8.1 (12.5) |
| Letter fluency | Exergame group | 19.8 (8.9) | Na | 20.9 (10.6) | 20.9 (11.2) |
|  | Aerobic group | 20.2 (9.1) | Na | 22.9 (9.5) | 23.5 (12.9) |
|  | Control group | 22.0 (11.8) | Na | 20.2 (12.0) | 21.7 (15.1) |
| Rule shift cards test | Exergame group | 7.8 (4.7) | 8.6 (4.7) | 8.3 (6.4) | 9.2 (5.5) |
|  | Aerobic group | 7.8 (3.7) | 8.7 (5.8) | 8.3 (5.6) | 7.6 (4.7) |
|  | Control group | 8.1 (5.1) | 8.6 (5.7) | 8.8 (6.2) | 8.9 (6.7) |
| Psychomotor speed | | | | | |
| Trail making test part A (sec) | Exergame group | 55.2 (51.2) | 52.7 (42.6) | 50.3 (42.0) | 55.8 (54.0) |
|  | Aerobic group | 45.9 (45.0) | 43.1 (42.0) | 37.7 (26.9) | 38.6 (40.2) |
|  | Control group | 48.2 (34.7) | 52.4 (47.2) | 63.6 (61.0) | 68.5 (73.4) |
| Stroop test word-reading (sec) | Exergame group | 41.4 (19.5) | 37.4 (10.5) | 37.3 (10.3) | 41.1 (16.4) |
|  | Aerobic group | 37.3 (13.7) | 36.7 (10.8) | 33.8 (8.5) | 35.7 (18.5) |
|  | Control group | 36.6 (12.7) | 41.3 (19.5) | 43.3 (22.6) | 49.7 (33.4) |
| Stroop test color-naming (sec) | Exergame group | 55.7 (25.6) | 50.2 (16.3) | 49.4 (17.9) | 55.0 (26.1) |
|  | Aerobic group | 45.1 (13.4) | 44.1 (14.0) | 42.8 (9.9) | 44.4 (22.8) |
|  | Control group | 52.9 (24.5) | 54.0 (24.3) | 56.7 (25.9) | 61.4 (36.1) |
| Episodic memory | | | | | |
| Location learning test displacement score trial 1-5 | Exergame group | 91.0 (35.0) | Na | 85.5 (41.7) | 96.1 (34.1) |
|  | Aerobic group | 95.3 (35.3) | Na | 96.6 (41.1) | 97.5 (41.3) |
|  | Control group | 97.6 (32.0) | Na | 107.1 (41.7) | 118.8 (51.2) |
| Location learning test displacement score delayed recall | Exergame group | 15.9 (8.5) | Na | 16.9 (11.2) | 18.5 (8.1) |
|  | Aerobic group | 18.8 (9.8) | Na | 19.1 (9.9) | 19.6 (10.5) |
|  | Control group | 18.3 (7.5) | Na | 20.0 (10.9) | 23.3 (10.8) |
| Working memory | | | | | |
| Digit span | Exergame group | 10.2 (2.8) | Na | 10.1 (2.9) | 9.6 (3.3) |
|  | Aerobic group | 11.1 (2.8) | Na | 10.8 (2.8) | 11.0 (3.2) |
|  | Control group | 11.2 (3.9) | Na | 10.8 (4.0) | 9.8 (3.6) |
| Spatial span | Exergame group | 8.6 (3.1) | Na | 8.3 (3.6) | 7.7 (3.8) |
|  | Aerobic group | 9.1 (3.1) | Na | 9.5 (3.2) | 9.4 (12.9) |
|  | Control group | 9.9 (3.6) | Na | 8.7 (3.6) | 9.3 (4.2) |

Abbreviations: Na=not applicable
